# Possible transmission of *Mycobacterium avium *subspecies *paratuberculosis *through potable water: lessons from an urban cluster of Crohn's disease

**DOI:** 10.1186/1757-4749-1-17

**Published:** 2009-09-23

**Authors:** Ellen S Pierce

**Affiliations:** 1 Spokane Valley, Washington, USA

## Abstract

A "cluster" of patients refers to the geographic proximity of unrelated patients with the same disease and suggests a common environmental cause for that disease. Clusters of patients with Crohn's disease have been linked to the presence of an infectious microorganism in unpasteurized milk and cheese, untreated water supplied by wells or springs, animal manure used as fertilizer for family vegetable gardens, and bodies of water contaminated by agricultural runoff.

*Mycobacterium avium *subspecies *paratuberculosis *(MAP) is the suspected cause of Crohn's disease. MAP causes a disease in dairy cows and other animals that is similar to Crohn's disease, called Johne's ('Yo-knees') disease or paratuberculosis. Dairy cows with Johne's disease secrete MAP into their milk and excrete MAP into their feces. MAP is present in untreated water such as well water, in bodies of water contaminated by agricultural runoff, and in unpasteurized milk and cheese.

The "treatment" of "tap" water to make it "drinkable" or "potable" by the processes of sedimentation, filtration and chlorination has little to no effect on MAP. MAP is so resistant to chlorine disinfection that such disinfection actually selects for its growth. Other subspecies of *Mycobacterium avium *grow in biofilms present on tap water pipes. Despite the documented presence of MAP in tap water and its probable growth on tap water pipes, clusters of Crohn's disease have not previously been described in relationship to tap water pipes supplying patients' homes.

This report describes three unrelated individuals who lived on the same block along a street in a midwestern American city and developed Crohn's disease within four years of each other in the 1960's. A common tap water pipe supplied their homes.

This is the first reported cluster of Crohn's disease possibly linked to fully treated drinking water, and is consistent with previously reported clusters of Crohn's disease linked to an infectious microorganism in water.

## Findings

A "cluster" of patients refers to the geographic proximity of unrelated patients with the same disease and suggests a common environmental cause for that disease. Clusters of patients with Crohn's disease have been previously reported in the literature [1-12]. They have been explicitly called clusters only when the individuals with Crohn's disease are unrelated, although studies of families with Crohn's disease also conclude that the disease is caused by an environmental factor [8-11] and specifically an infectious one [9,11]. These previously reported clusters of Crohn's disease have been linked to the consumption of unpasteurized milk [9], the consumption of unpasteurized cheese [10], drinking untreated water supplied by wells [1,10] or springs [3,9], the use of animal manure as fertilizer for family vegetable gardens [10], and swimming in [2] or inhaling aerosolized droplets from [12] bodies of water contaminated by agricultural runoff.

*Mycobacterium avium *subspecies *paratuberculosis *(MAP) is the suspected cause of Crohn's disease [13,14]. MAP causes a disease in dairy cows and other animals that is similar to Crohn's disease, called Johne's ('Yo-knees') disease or paratuberculosis [15]. Dairy cows infected with MAP secrete the organism in their milk [16] and the process of pasteurization does not completely kill the organism [17]. Cows infected with MAP also excrete the organism in their feces [18], and cow manure is commonly spread onto agricultural land, the runoff of which contaminates many bodies of water [19,20]. MAP is present in pasteurized milk [17,21], unpasteurized or "raw" milk [22], cheese made from both pasteurized [23,24] and unpasteurized [25] milk, untreated water [26] such as well water, and bodies of water contaminated by agricultural runoff [12].

While the presence of MAP in milk and products made from milk has been intensively investigated, the role of water in transmitting MAP to humans has not been as carefully studied. The "treatment" of "tap" water to make it "drinkable" or "potable" consists of combinations of sedimentation, filtration, and disinfection, usually by chlorination [27]. These normal water treatment processes have little to no effect on mycobacteria, including MAP [28,29]. In particular, as a member of the *M. avium *complex (MAC) group of microorganisms [30], MAP is so resistant to chlorine and other disinfectants [31] that such disinfection actually selects for its growth [32]. MAP organisms survive and grow in waterborne protozoa and are even more resistant to chlorine disinfection in such protozoa [33,34]. One of the subspecies of the MAC complex also flourishes in biofilms present on tap water pipes [35].

Because of the survival and possible selection for and growth of MAP in tap water by this variety of mechanisms, it is more likely that MAP is frequently transmitted to humans through drinking water rather than pasteurized milk. Organisms from the genus *Mycobacterium *[36], the *M. avium *complex [37,29] and MAP in particular [38,39] have been identified in drinking water.

If MAP organisms survive and flourish in tap water pipes, they will be present in tap water at higher concentrations the more distant the pipes are from the water treatment facility [35]. The presence of MAP in higher concentrations in tap water pipes farther away from water treatment facilities would explain the superficially paradoxical epidemiology of Crohn's disease, which until recently [40] has been more common in urban areas (farther away from the cow and other animal feces that are the source of MAP) than rural ones [41].

Despite the documented presence of MAP in drinking water and the generally higher prevalence of Crohn's disease in urban as compared to rural areas, clusters of Crohn's disease have not been reported in relationship to particular tap water pipes supplying patients' homes. This is the first such report.

It was brought to the author's attention that there was a cluster of three individuals with Crohn's disease along a street in a midwestern American city. The city receives tap water fully treated from a regional water system. All three individuals are Jewish. They are not related to one another. They all developed Crohn's disease after moving to the street. They did not have any social contact with one another and did not spend any time in one another's homes.

**Figure 1:**
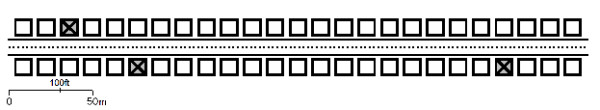
**Street Diagram**. The three Crohn's disease cases lived in the three houses marked with an X. The woman who had Crohn's disease before her move to the street lived in one of the unmarked houses. A six (6) inch tap water pipe in the middle of the street supplies houses on both sides. Water in this pipe travels in a continuous loop in both directions, with no dead ends in the system.

Case 1 moved to one of the three houses marked X in Figure 1 in 1957, when she was 8 years old, and lived there until 1967. She was diagnosed with "regional enteritis" in 1966 at age 17 after "feeling faint" in the stands at her high school graduation, and underwent a single surgery shortly thereafter. Her continuing "cramps and diarrhea" improved after the birth of each of her three children, and she is asymptomatic now.

Case 2 moved to another of the three houses marked X in Figure 1 in 1958, when he was 36 years old, and lived there until his death in 1978 from unrelated causes. He was diagnosed with "ileitis" in 1964 at age 42 after a sudden illness that was originally thought to be appendicitis. He underwent a second small bowel surgery 7 years later.

Case 3 lived on the street, in another of the 3 houses marked X in Figure 1, from her birth in 1963 until 1981. She was diagnosed with Crohn's disease in 1981, with multiple strictures throughout the ileum, after a 6-month history of abdominal pain and vomiting. She dates her Crohn's disease to 1968 based on her memory of early satiety and delayed growth and puberty.

Estimating 5 persons per household in each of the 50 houses in Figure 1, the prevalence of Crohn's disease on this block was 3/250, or 120/10,000, over 3 times the prevalence of Crohn's disease in high-incidence countries [42].

This is the first reported cluster of individuals with Crohn's disease possibly linked to treated water, and is consistent with previously reported clusters of Crohn's disease linked to an infectious microorganism in water. The length of time between living on the street and the development of Crohn's disease (8 years for case 1, 6 years for case 2, and 5 years for case 3) is consistent with the previously reported latent period between exposure to an infectious microorganism in water and the development of Crohn's disease [2,3,7].

The previously reported clusters of Crohn's disease associated with water have been linked to swimming in polluted ground water [2], and to drinking untreated well [1] or spring [3,9] water. Only one cluster has been linked to possibly "polluted" tap or potable water [7]. This is the first cluster of Crohn's disease linked to clean, unpolluted, fully treated drinking water. As emphasized above, the normal methods of treating water for human consumption are completely ineffective against MAP and other environmental mycobacteria, and currently used chlorine disinfection actually selects for the growth of MAP and other MAC organisms. It is therefore possible that MAP organisms, like other environmental mycobacteria, were present in the fully treated drinking water supplying the cases' homes [43].

Another woman, diagnosed with large bowel Crohn's disease a "year or two" before her move to the street, lived on the street from 1961, when she was 28 years old, to 1971, in one of the unmarked houses in Figure 1. She is not part of the cluster, since she moved to the street after being diagnosed with Crohn's disease. This woman had Crohn's disease in the colon, whereas the three cases in this cluster all had small bowel disease. This suggests that different strains of MAP might be responsible for the different anatomic locations of Crohn's disease.

The fact that all three cases are Jewish, suggesting genetic similarities, does not rule out an infectious cause for their Crohn's disease. Multiple genetic polymorphisms are associated with an increased or decreased risk of developing tuberculosis and leprosy, the other major mycobacterial diseases of humans [44]. Polymorphisms of the NOD2 gene are associated with an increased risk of Crohn's disease in humans [45] and Johne's disease in cattle [46]. NOD2 is involved in the immune response to intracellular pathogens [47], particularly intracellular pathogens of the genus *Mycobacterium *[48,49], and NOD2 specifically recognizes MAP [50].

## Competing interests

The author declares that they have no competing interests.

## Consent

Written informed consent for publication of this report and the accompanying figure was obtained from cases 1 and 3, the widow of case 2, and the woman who had Crohn's disease before her move to the street.
